# Decellularization of Human Digits: A Step Towards Off-the-Shelf Composite Allograft Transplantation

**DOI:** 10.3390/bioengineering12040383

**Published:** 2025-04-03

**Authors:** Michelle E. McCarthy, Irina Filz von Reiterdank, Oliver H. Parfitt van Pallandt, McLean S. Taggart, Laura Charlès, Korkut Uygun, Alexandre G. Lellouch, Curtis L. Cetrulo, Basak E. Uygun

**Affiliations:** 1 Center for Engineering for Medicine and Surgery, Department of Surgery, Massachusetts General Hospital, Harvard Medical School, Boston, MA 02114, USA; 2 Shriners Children’s Boston, Boston, MA 02114, USA; 3 Vascularized Composite Allotransplantation Laboratory, Massachusetts General Hospital, Harvard Medical School, Boston, MA 02114, USA; 4 Department of General Surgery, Beth Israel Lahey Hospital and Medical Center, Burlington, MA 01805, USA; 5 Department of Plastic, Reconstructive and Hand Surgery, University Medical Center Utrecht, 3584 CX Utrecht, The Netherlands; 6 Department of Plastic, Reconstructive et Aesthetic Surgery, Hôpital Paris Saint-Joseph, 75674 Paris, France; 7 Innovative Therapies in Haemostasis, INSERM UMR-S 1140, University of Paris, 75006 Paris, France; 8 Division of Plastic and Reconstructive Surgery, Cedars Sinai Hospital, Los Angeles, CA 90048, USA

**Keywords:** decellularization, vascularized composite allografts, digits, human, transplantation, rejection

## Abstract

The field of reconstructive surgery faces significant challenges in addressing limb loss and disfigurement, with current organ preservation methods limited by short storage times. Decellularization offers a promising solution for generating engineered alternatives for reconstructive surgery by removing cellular material while preserving the extracellular matrix (ECM) and providing scaffolds for tissue regeneration. In this study, we developed a robust protocol for decellularizing whole digits from long-term freezer storage, achieving the successful removal of cellular material with intact ECM. Digit angiography confirmed the preservation of vascular integrity, facilitating future perfusion for recellularization. Quantitative analysis revealed significantly lower DNA content in decellularized tissues, indicating effective decellularization. Furthermore, extracellular matrix analysis showed the preservation of collagen, elastin, and glycosaminoglycans (GAGs) contents. Histological examination confirmed the reduction in cellularity and maintenance of tissue architecture in decellularized digits. Mechanical strength testing of decellularized digit tendons proved consistent with that of native digits. Our findings highlight the potential of decellularized digits as versatile platforms for tissue engineering and regenerative medicine. Moving forward, further optimization of protocols and collaborative efforts are essential for translating these findings into clinical practice, offering innovative solutions for reconstructive surgery and limb transplantation.

## 1. Introduction

With 100,000 digit and 185,000 limb amputations taking place yearly in the United States alone and only 33% being candidates for replantation, the field of reconstructive surgery is facing serious limitations [1]. Despite decades of research, a critical bottleneck is that current organ preservation times are limited to a few hours of storage [2]. Especially on battlefields and with other remote or severe trauma patients, limited preservation time inhibits the salvageability of organs, resulting in life-altering amputations and disfigurement. Vascularized Composite Allografts (VCAs) provide patients with an esthetic and functional reconstruction of their severed or disfigured body parts. However, VCAs carry a high risk of rejection with numbers as high as 89% of patients undergoing acute rejection episodes [3,4], not to mention comorbidities due to lifelong systemic immunosuppression and even mortality [5]. Tissue engineering offers tools to recreate potentially non-immunogenic tissues replacing lost autologous tissue in patients. One such tool, decellularization of tissues, aims to remove all genetic material while preserving the extracellular matrix (ECM), which serves as a scaffold for recellularization [6]. By removing all immunogenic material while keeping the structure intact, the decellularized matrix scaffold can be repopulated with the recipient patient-derived or other non-immunogenic cells.

In solid organ tissue engineering, pioneering research in rat livers showed decellularization and recellularization, achieving successful results of more than 90% rat liver tissue recellularization [7,8,9,10,11]. The application of this approach is relatively recent in VCAs [12], but decellularization of porcine fasciocutaneous flaps [13], human face [14,15,16], ear [16,17], penile [18], and limb grafts [19,20], as well as non-human primate [19], porcine [17,21,22], and rodent models [15,23,24], have been demonstrated. Moreover, the potential use of decellularized tendons as a replacement for tendon transfer surgery could be of interest, whereby the maintenance of their mechanical properties when compared to that of native digit tendons is of importance [25,26]. However, the decellularization of more complex VCAs, such as whole digits and limbs, has presented challenges due to their intricate structure and varied tissue types. Recellularization efforts have focused on repopulating the decellularized scaffolds with endothelial cells, fibroblasts, myoblasts, adipose-derived stem cells, and mesenchymal stem cells [12]. Although achieving complete and functional recellularization with successful transplantation remains a significant hurdle, cellular engraftment and functional integration could be achieved in some cases with limited success [20,24].

Due to the complexity of VCAs containing multiple tissue types, the vast network of capillaries in muscle tissue, and the difficulty of obtaining fresh VCA tissue from donors [27], studies on human VCAs are limited. Establishing a protocol for decellularization and recellularization of human digits would provide a more accessible and replicable model for high-throughput research, while maintaining the various tissue types of VCAs within the model. Using organs stored with traditional subzero freezing methods could greatly increase the availability of discarded tissues, providing researchers and clinicians with ample VCA material. This would facilitate the optimization of decellularization and recellularization protocols, ultimately advancing the field. Clinically, non-immunogenic digit grafts could provide patients with severe digital trauma a safe alternative, preserving both function and esthetics.

The objective of this study was to establish a robust, non-destructive decellularization protocol for human VCA models using conventionally stored human digits. By developing this protocol in a clinically relevant and high-throughput manner, this work aimed to advance research towards the development of engineered alternatives for improved VCA transplantation.

## 2. Materials and Methods

### 2.1. Human Digit Procurement

Eight upper extremities of unidentified human donors (male, 77 years old, right extremity; male, 79 years old, left extremity; male, 81 years old, left extremity; male, 84 years old, right extremity; male, 87 years old, right extremity; female, 80 years old, right extremity; female, years old, right extremity; female, 85 years old, left extremity) were obtained under MGH IRB approval (2019P002672) and stored at −20 °C for 4–6 months. A total of 29 human digits (digits 2–5 of each upper extremity) were procured simultaneously by multiple surgeons, after overnight thawing. V-shape incisions are made on the ventral and dorsal side. Subsequently, digital arteries were identified, skeletonized, and ligated on the ventral side and digital veins underwent a similar procedure on the dorsal side. Arteries and veins are cannulated with 18G angiocatheter and secured with 4-0 silk suture. Next, 18 digits were subjected to the decellularization protocol as described below and 11 digits were used as native controls.

### 2.2. Perfusion Decellularization

**Figure 1:**
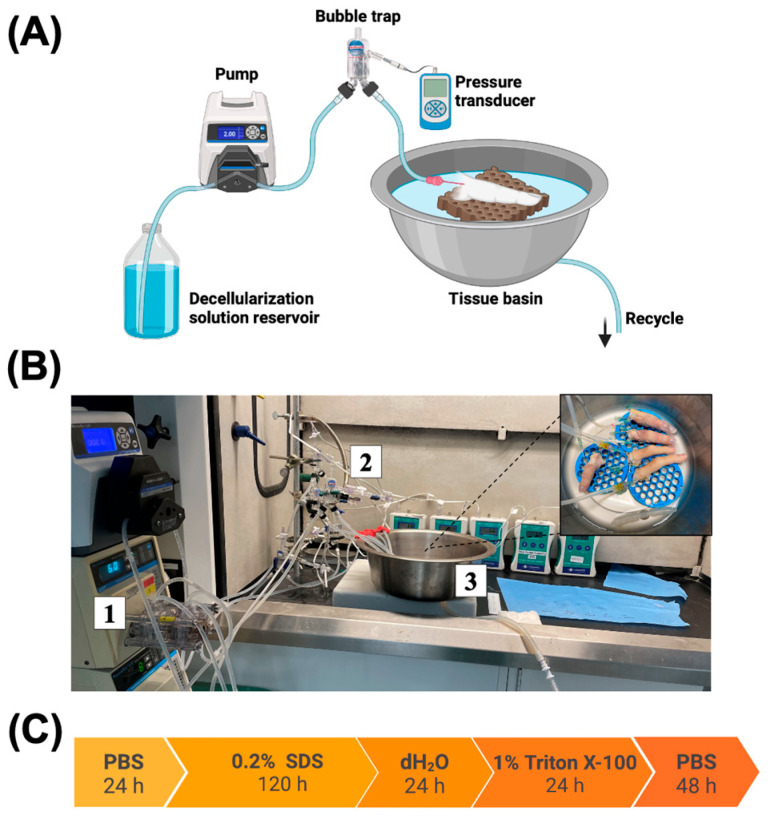
Human digit decellularization. (**A**) Schematic representation of perfusion setup. (**B**) Photographic image of perfusion system. Flow-controlled, continuous pump (1) runs perfusate to bubble trap and pressure monitor (2) to maintain 40–60 mmHg pressures before entering tissue basin in which the are submersed in perfusate (3). (**C**) Decellularization protocol shows solutions used and their durations.

Solutions were circulated through the digits using Masterflex^®^ L/S^®^ digital drive equipped with an Easy Load^®^ II pump head (Cole-Parmer, Vernon Hills, IL, USA) with Masterflex platinum-cured silicone tubing, tubing size 16 (Cole-Parmer, Vernon Hills, IL, USA). A pressure transducer (PT-F, Living Systems Instrumentation, St Albans City, VT, USA) was connected to the inlet tubing close to the angiocatheter (BD Angiocath 18G, Franklin Lakes, NJ, USA) in the digital artery during perfusion (Figure 1A,B). Vascular resistance (flow and pressure) was monitored continuously; flow rate was manually adjusted to reach a target pressure of 40–60 mmHg. A bubble trap (Radnoti, Covina, CA, USA) was placed before the solution entered the tissue to eliminate any air bubbles. Closed loop perfusion was performed at room temperature (21 °C). The perfusion setup was designed to process five digits in parallel. Eighteen digits were decellularized through perfusion with solutions based on the optimization study performed by Lupon et al. on porcine fasciocutaneous flaps [13]. Phosphate-buffered saline (PBS) (Sigma-Aldrich, St. Louis, MO, USA) was perfused for 1 h to wash out the vasculature, followed by 0.2% sodium dodecyl sulfate (SDS) (Sigma-Aldrich, St. Louis, MO, USA) as an ionic detergent for 120 h. Following SDS perfusion, the digits were perfused with deionized water for 24 h, then 1% Triton X-100 (Sigma-Aldrich, St. Louis, MO, USA) as a non-ionic detergent for 24 h. Finally, the digits were perfused with PBS for 48 h to washout any remaining detergent solutions (Figure 1C).

### 2.3. Digit X-Ray with Contrast

The vascular integrity of digits before and after decellularization was assessed using X-ray with contrast. Omnipaque (iohexol, GE Healthcare, Anaheim, CA, USA) contrast agent was slowly injected into the digital artery via the previously placed angiocatheters. The injection rate and volume were tailored to ensure optimal opacification of the digital vasculature, and to minimize background image noise. Immediately following contrast agent injection, standard X-ray imaging was performed using a MinXRay HF100 ultralight imaging system (Northbrook, IL, USA). Images were acquired with a standardized dorsal–ventral view (62 kV, 1.20 mA/s power parameters at 28 cm distance above the specimen) to visualize the arterial anatomy of the digit in detail.

### 2.4. DNA Quantification Assay

DNA extraction from decellularized digit tissue samples of skin, vessel, muscle, nerve, and bone was performed using a DNeasy Tissue Kit (Qiagen, Hilden, Germany) according to the manufacturer’s protocol for purification of total DNA from animal tissues (Spin-Column Protocol). Briefly, 25 mg of wet tissue sample was resected from decellularized or native human digits, and three technical replicates were carried out for each tissue type and extraction was performed according to the manufacturer’s instructions. Native and experimental digits were procured in a similar fashion and randomly allocated to their groups. Experimental digits underwent the decellularization protocol while native digits immediately underwent dissection post-procurement. The concentration of the extracted DNA was determined by spectrophotometric absorption using a Nanodrop spectrophotometer (Nanodrop One, Thermo Fischer Scientific, Waltham, MA, USA). Before starting DNA content measurements, the NanoDrop was blanked using the DNeasy kit elution buffer. Following this, 1 uL aliquots of the extracted DNA samples were measured using an absorbance ratio of 260/280 nm. Based on the measured absorbance, the absolute concentration (ng/μL) of DNA was calculated using the empirically derived conversion factor provided by the NanoDrop software (2.9.07) and reported as ng of DNA in mg of wet tissue.

### 2.5. Biochemical Assays for Quantification of ECM Components

#### 2.5.1. Collagen Assay

The content of collagen in the decellularized digit tissues of skin was determined using a total collagen assay kit (MAK322, Sigma Aldrich, St. Louis, MO, USA) following the manufacturer’s protocol. Samples of 20 µg from each tissue were collected and were run with three technical replicates. The samples were first homogenized in saline via sonication. These tissue samples were then digested by adding 30 μL of Master Reaction Mix containing assay buffer and digestion enzyme and incubated for 1 h at 37 °C in black flat bottom 96-well plates. Following this, 40 μL of dye reagent was added to all wells, followed by incubation at 37 °C for 10 min. Lastly, 8 μL of the Developer Reagent was added to all wells and incubated at 37 °C for 10 min. The fluorescence of the wells was read at 375 nm excitation and 465 nm emission wavelengths using SpectraMax iD3 multiwell plate reader (Molecular Devices, San Jose, CA, USA). A collagen standard curve range (0–50 μg/mL) was also measured according to the kit manual. Collagen content (μg/mg wet weight) of the tissue samples was determined by comparing the values measured for absorbance to those of the known concentrations in the collagen standard curve made using the kit.

#### 2.5.2. Glycosaminoglycan (GAG) Assay

Quantification of glycosaminoglycans (GAGs) was performed using a Total Glycosaminoglycans Assay Kit (Colorimetric, ab289842) (Abcam Limited, Cambridge, UK). Sample preparation consisted of 200 mg wet tissue collected from the skin tissue of native and decellularized digits with three technical replicates from each digit. First, 2X volume of ice-cold GAG Assay Buffer is added to each sample. They are then homogenized using a Dounce Tissue Homogenizer. Next, the samples were centrifuged at 12,000× *g* and 4 °C for 20 min and the supernatant collected. Then, 10–50 μL of the supernatant for each sample was added to a clear bottom 96-well plate. In parallel, a standard curve was prepared in accordance with the kit manual by adding 0, 2, 4, 6, 8, and 10 μL of GAG Standard (1 mg/mL) into the 96-well plate to generate 0, 2, 4, 6, 8, and 10 μg of GAG Standard/well, respectively. Wells for both the sample and standard were adjusted to a volume of 100 μL using GAG Assay Buffer. Finally, 200 μL of GAG Probe was pipetted carefully into all wells including GAG standard, samples, and background control, and the plate was incubated for 2 min at room temperature. The absorbance was then measured using a SpectraMax iD3 multiwell plate reader (Molecular Devices, San Jose, CA, USA) at 400 nm. The absorbance values measured for the samples were then compared against the results plotted for the known concentrations of the GAG standard curve to derive their respective GAG content (μg/mg wet weight).

#### 2.5.3. Elastin Assay

Quantification of elastin content was performed using a Fastin Elastin Kit (Biocolor Life Science Assays, Carrickfergus, UK) following the manufacturer’s recommendations. Briefly, 20 mg of skin tissue samples were collected from native and decellularized digits. Three technical replicates were collected from each digit. The samples were digested with 0.25 M oxalic acid at 100 °C for 60 min. Th samples were then allowed to cool to room temperature and subsequently centrifuged at 13,000× *g* for 10 min. The remaining tissue was then digested with 0.25 M oxalic acid and centrifuged once more. Supernatants were then pooled and analyzed according to the manufacturer’s instructions using a SpectraMax iD3 multiwell plate reader (Molecular Devices, San Jose, CA, USA) at 513 nm.

#### 2.5.4. Histology

After decellularization, biopsies were taken of skin, muscle, tendon, vasculature, and bone and histological analysis was performed at the National Institutes of Health P30 Histology and Histomorphometry Core at the Center for Musculoskeletal Research at Massachusetts General Hospital. Briefly, the biopsies were fixed in formalin and processed for histopathological examination. Tissues containing bone were decalcified by submersion in 20% ethylenediaminetetraacetic acid (EDTA) solution for 4–6 weeks prior to fixation in ethanol 70%. The slides were embedded in paraffin and sectioned and stained with hematoxylin and eosin (H&E(Abcam, Cambridge, MA, USA) to assess general tissue architecture; Verhoeff–Van Gieson for elastin structure and composition, Masson’s Trichrome for collagen distribution and density, and Alcian Blue for distribution and structure of mucopolysaccharides and GAGs. A blinded evaluation by a pathologist was performed for all biopsy samples.

#### 2.5.5. Mechanical Testing of Tendons

Tensile strength of native (n = 10) and decellularized digit tendons (n = 12) was measured using the Instron 5500 Universal Testing Machine at the Department of Polymer Science and Engineering, University of Massachusetts Amherst. Digit tendons resected include the digital extensors and the superficial and deep flexors. Tendon specimens were first gripped between the upper and lower clamps of the Instron machine. Then, using calipers, the distance between grips, or initial gauge length ($L_{0}$), and the cross-sectional area ($A_{0}$) were measured and used as input prior to each test. The machine then applied an increasing force (F), recorded via its load cell (high precision force-sensor) while pulling the clamped ends of the tendons away from each other, resulting in an elongating change in tendon length (∆L), up until rupture. From these continuous measurements, % tensile strain ε, where $\varepsilon \;=\frac{∆L}{L_{0}}\times 100\;$, and tensile stress σ, where $\sigma =\frac{F}{A_{0}}$ measured in MPa were recorded. From these data, the tendon elastic modulus (stiffness), or Young’s Modulus E where $E=\frac{\sigma }{\varepsilon }$, was estimated (Figure S2) [28,29]. To calculate the estimated value of the Young’s Modulus for each tendon, a Python (Version 3.9.6) script specified to the output of the Instron 5500 Universal Testing Machine used was implemented (Figure S3). This script iterates through data values of % tensile strain and tensile stress (150–800 data points per tensile test) and returns a complete plot per tendon, highlighting the region of elastic modulus, as well as the most positive value of this region pertaining to the Young’s Modulus.

#### 2.5.6. Dynamics Function

After decellularization, the range of motion of the digits was assessed visually by manually retracting the superficial and deep flexor tendons and the extensor tendons of the digits.

#### 2.5.7. Statistical Analysis

Continuous variables are presented as means ± standard deviation (SD), unless otherwise stated. To assess the significance of differences between two independent groups, Student’s *t*-test for independent samples was employed. Outliers were excluded using the ROUT method (Q = 1%) [30]. All statistical analyses were performed using Prism 9 for Mac OSX (GraphPad Software, La Jolla, CA, USA). A significance level of 0.05 was used to determine statistical significance.

## 3. Results

### 3.1. Removal of Cells from Digits After Decellularization

**Figure 2:**
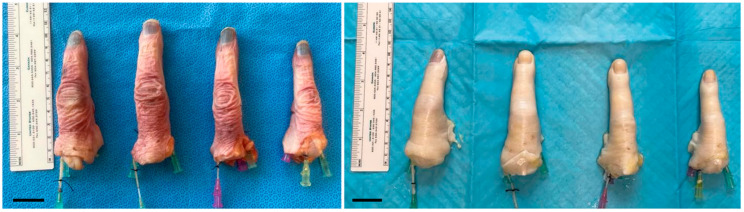
Representative gross morphology images of human digits before (**left**) and after (**after**) decellularization. Scale bars represent 1 inch.

A total of 18 digits were decellularized following a perfusion decellularization protocol that was optimized for VCAs [13]. At the end of the protocol, decellularized digits exhibited notable changes in gross appearance compared to their native counterparts (n = 11) (Figure 2). The decellularized digits appeared translucent and devoid of pigmentation, with a pale white coloration. Furthermore, there was an increase in overall size and bulkiness observed in the decellularized digits, likely due to the increase in interstitial fluid.

**Figure 3:**
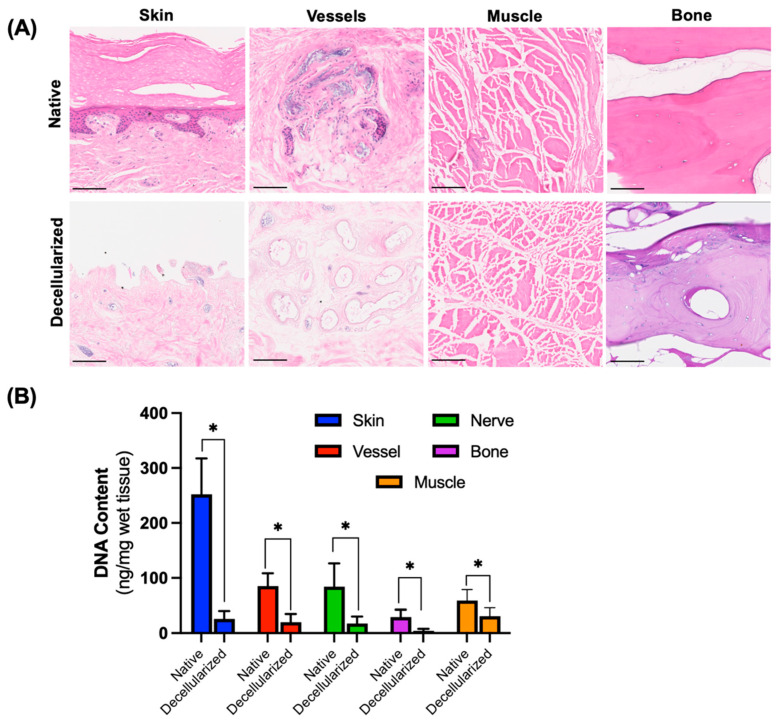
Removal of cells from human digits. (**A**) Microscopic histology images (H&E). (**B**) DNA content of different tissues within native and decellularized digits. Scale bar represents 100 µm. * *p* < 0.05 by Student’s *t*-test, n = 4.

Microscopically, decellularized digits demonstrated significantly less cellular content compared to native digits in different tissue parts based on H&E staining (Figure 3A). Specifically, the epidermal layer was completely removed from the skin and distinct nuclear structures in all dermal layers were absent. Capillaries in the dermis of the decellularized samples show a lack of cellularity and intact nuclear structures. The cellular integrity and adhesion were lost, but residual nuclear debris was found in the capillary lumen although differing from the intact and cellularly rich native samples. The intimal layers of the vasculature remained distinct and intact. The ligament sections were devoid of any nuclear stain after decellularization. The fibers showed some loss of orientation compared to the linear longitudinal orientation of fibers in native samples. While sections from the nailbed and the bone were not completely clear of remnant nuclear stain, the extent of staining was notably less in the decellularized than in the native digit sections. In bone tissue, preservation of several nuclei is noted; however, there was a clear reduction compared to the control, with nuclei largely absent while the bone matrix is generally preserved. The removal of cells was confirmed through quantification of the DNA content in the tissues (Figure 3B). It was found that DNA removal was 89.7% in the skin, 76.9% in the vessels, 79.3% in the nerves, and 85.5% in the bone as a result of decellularization. DNA content was reduced by 47.5% in the muscle sections of the digits. The reduction in the decellularized tissue DNA content was statistically significant and was ≤30 ng/mg wet tissue for all tissue types.

### 3.2. Preservation of Extracellular Matrix Components

**Figure 4:**
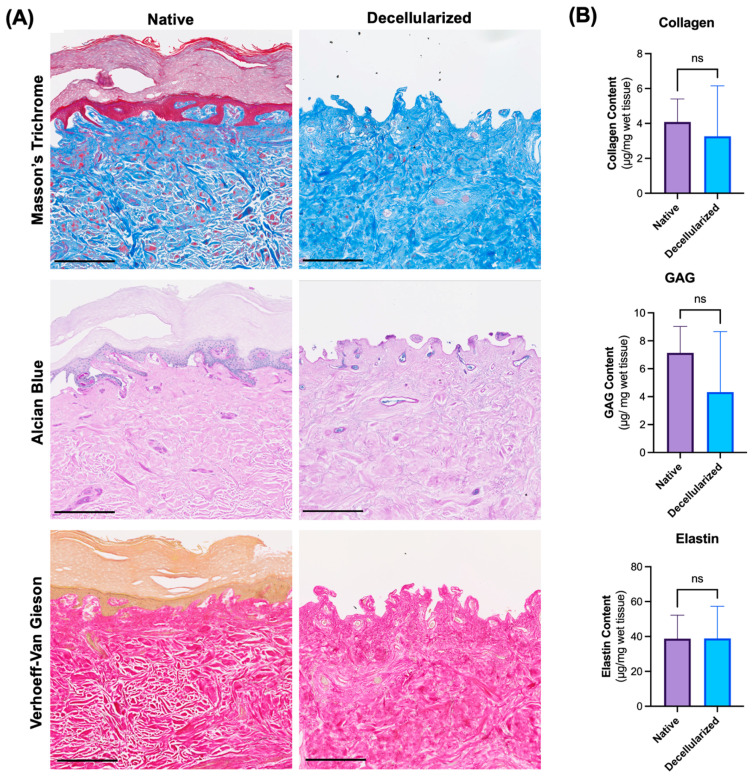
Extracellular matrix components in skin tissue of human digits. (**A**) Histological sections of native and decellularized digits stained with Masson’s Trichrome, Alcian Blue, and Verhoeff–Van Gieson stain. (**B**) Biochemical quantification of collagen, GAG, and elastin contents of skin in native and decellularized digits. Scale bar represents 250 µm. ns—not significant by Student’s *t*-test.

In order to assess whether the ECM in the digits is preserved after decellularization, histological sections of the skin, vessels, muscle, and bone were stained with Masson’s trichrome for collagen, Alcian Blue for GAGs, and Verhoeff–Van Gieson for elastin and the skin matrix was analyzed biochemically (Figure 4). Due to the high amount of tissue required to assess ECM components, as described in the Section 2, quantitative analysis was restricted to skin tissue. However, in the digit, skin tissue is the most challenging to decellularize due to its high cell count and microcapillary network, while being the most relevant in terms of immunogenicity. Therefore, we believe it is a relevant representation of the ECM structure in the other tissues of the digit.

The basic architecture of the dermal layers, although less defined, was maintained in the decellularized samples. Elastin and collagen fibers were preserved in both the control and decellularized samples, showing some disorganization and fragmentation in the decellularized group (Figure 4A). Correspondingly, GAGs showed maintenance of intensity in the decellularized samples compared to the native samples. While some changes in ECM components were found, structural integrity appeared relatively preserved. The main alterations found were the loss of organization of ECM components such as elastin fibers, collagen fibers, and GAGs. Assessment of the decellularized tissues showed little variability in histological outcomes between replicates. Representative histological images of vessel, muscle, and bone tissue are displayed in Figure S1. Quantitative analysis revealed no significant differences in the quantities of collagen, elastin, and GAG contents between the skin of the native digits and decellularized digits (Figure 4B).

These findings indicate that the decellularization process did not significantly alter the ECM composition, as evidenced by the comparable levels of collagen, elastin, and GAG contents in the skin of the native and decellularized digits. This preservation of extracellular integrity further supports the suitability of decellularized digits as scaffolds for tissue engineering and regenerative medicine applications.

### 3.3. Maintenance of Vascular Network

**Figure 5:**
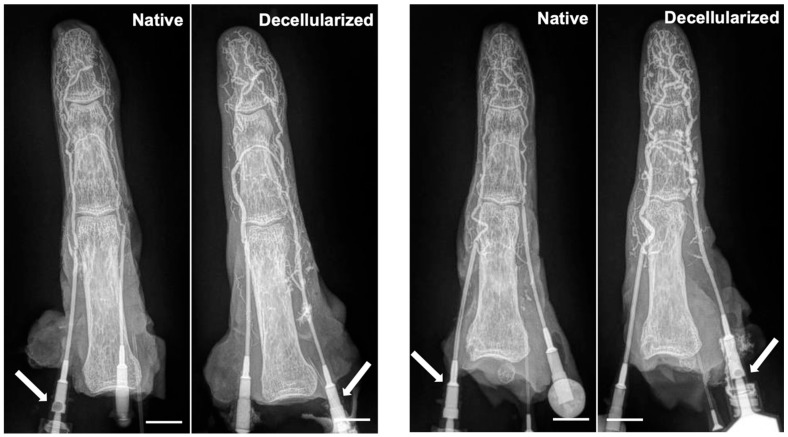
Digital X-ray with contrast images of native and decellularized digits. Two representative digits (**left** and **right**) are shown. Scale bars represent 1 cm. Arrow depicts artery through which contrast fluid was injected.

Digit angiography performed before and after decellularization demonstrated the maintenance of the vascular network within the digits (Figure 5). Contrast-enhanced imaging demonstrated a robust and patent vascular system, with no discernible disruption in arterial or venous flow patterns post-decellularization. Gross analysis of the angiographic images indicated the presence of well-defined arterial branches supplying the digits, maintaining their characteristic branching patterns and perfusion territories. Furthermore, venous drainage pathways remained intact.

### 3.4. Mechanical Testing of Tendons

**Figure 6:**
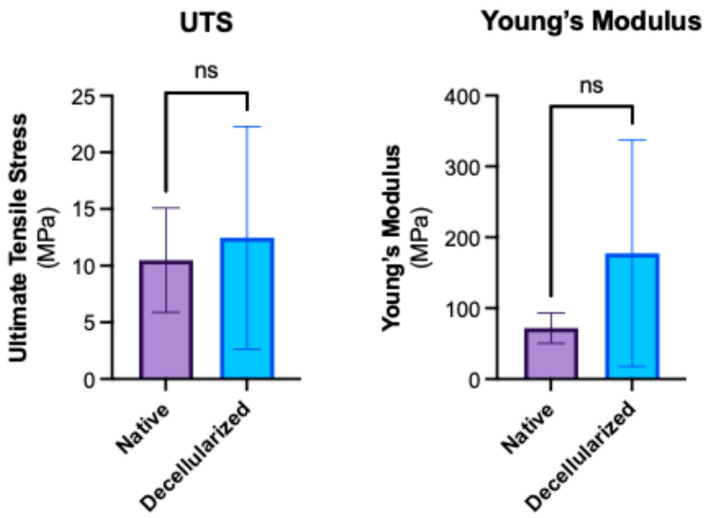
Mechanical strength testing of decellularized digit tendons. Quantification of tensile stress maximum values and calculated Young’s modulus values of digital tendons from native and decellularized digits. ns—not significant by Mann-Whitney test.

Tensile strength testing showed that there was no significant difference between the ultimate tensile stress (*p* = 0.9754) and stiffness (Young’s Modulus) (*p* = 0.1229), between the experimental and control group (Figure 6). The results separated by the different tendon types are presented in Figure S4, and similarly show no significant differences between the groups.

### 3.5. Dynamic Function

After decellularization the range of motion of the digits was assessed as a measure of potential intrinsic function. Video S1 shows, in sequence, the isolated range of motion of the (1) superficial flexor, (2 and 4) extensor, and (3) deep flexor tendon. It is demonstrated that the tendons have a near full range of motion and show isolated function, despite the increase in interstitial fluid.

## 4. Discussion

Tissue-engineered composite allografts may provide reconstructive alternatives for patients with severe tissue defects by replacing like with like, while addressing limitations of current VCA transplantation such as the short preservation time and high rejection rates. By removing cellular components while preserving the ECM scaffold, decellularized digits serve as biocompatible scaffolds for cell repopulation and tissue regeneration. In contrast to solid organs, VCAs consist of multiple tissue types, thereby adding complexity to achieving de- and recellularization. Therefore, simplified high-throughput models are necessary to make progress in this field. While various animal models have been developed in both small and large animals [13,15,17,19,21,22,23,24], a model using human tissue would be preferential to limit future translational hurdles. In this study, we demonstrate the successful removal of cellular material by perfusion of the vascular network and imbibition following a protocol developed for fasciocutaneous flap decellularization [13]. Further, we demonstrate the maintenance of tissue architecture in decellularized human digits that were stored at −20 °C for over 4 months, showing their potential as non-immunogenic platforms for tissue engineering applications.

Previous studies using human VCA models have decellularized a human face [14,16], ear [16], penile scaffolds [18], and an upper extremity [19], which were either fresh or stored at 4 °C or flash frozen for less than 24 h. By making use of conventionally stored human VCA tissue in this study, the method aids in overcoming logistical hurdles related to organ donation. Furthermore, the use of functional subunits, namely digits, allows for multiple replicates per extremity donation, which is especially important in grafts that are hard to come by such as upper extremities. The first decellularization study on a full human extremity [19] highlighted the challenges of obtaining human specimens and the length of the decellularization protocol (60 days). By multiplying the use of each donated human arm by using digits as a model, protocol optimizations can be made prior to moving to larger models such as full upper extremities. Furthermore, in contrast to ear and penile grafts, digits contain multiple tissue types, making them a relevant VCA model. Meanwhile, due to the limited presence of muscle, certain complexities of large tissue decellularization, such as compartment syndrome [19,20], can be avoided. For the same reason, the relatively small size of the digits lends itself to early recellularization studies. As outlined by Adil et al. [31], many factors need to be investigated to enable successful recellularization. These include defining the required cell population(s) to achieve functional grafts, establishing a biocompatible ECM scaffold for in vivo use, and developing an ex vivo bioreactor for functional maturation of the graft during the recellularization process. All of these can be investigated using human digits prior to moving to larger models.

Several groups have investigated the decellularization of composite tissues using both small and large animal models [20,22]. Here, as well as in a study on a human extremity [19], maintenance of vascular outflow was critical for achieving decellularization, preservation, and subsequent recellularization [20]. Importantly, this study showed that all digits maintained vascular outflow during the entire protocol, eliminating this challenge. In addition, this study showed a significant reduction in the DNA content of skin tissue. In combination with maintained ECM structures by quantitative and qualitative analysis, as well as the vascular network as shown on X-ray, and dynamic function, these grafts provide a promising scaffold for future recellularization studies. Similar decellularization results were found in the protocol used by Jank et al., though it was in rat and nonhuman primate forearms. Recellularization was also performed using techniques that could work well for translation to human models, such as our proposed human digits.

While SDS and Triton X-100 have been widely used for decellularization purposes, their destructive effects on ECM structures have been demonstrated [32]. For instance, alterations in collagen and elastin macromolecules were found in venous valved conduits that underwent SDS decellularization, resulting in collagen fibril merging and structural disruption, potentially affecting mechanical properties and long-term graft integration [33]. Although our ECM analysis indicated preservation of gross ECM composition presumably due to the use of low SDS concentrations [13], subtle disruptions at the ultrastructural level likely occurred. Future investigations using detailed imaging, such as electron microscopy, would further clarify these structural impacts and inform improvements to the decellularization protocol.

Limitations of this study are twofold. First, while DNA content was significantly lower in the decellularized group, some nuclei and cell debris were found in histology, which can be immunogenic once transplanted. Extending the perfusion protocol could provide a solution to this. However, in the literature, it was proposed that complete cell removal may not be necessary [12], and that achieving a chimeric graft consisting of donor and recipient cells may be sufficient to avoid graft rejection. From a technical perspective, it was not possible to do a direct comparison of the DNA content levels to the threshold recommended by Crapo et al. [34], as this threshold refers to dry weight rather than wet weight, which was used in this study. Moreover, Lopera Higuita et al. [35] showed that besides DNA content, ECM components can also contain antigenic content, which can cause an immune response. Second, a downside of using human digits as a model for decellularization is the limited availability of certain tissue types for analysis and batch-to-batch variation. This does not inhibit skin assessments; however, vascular, nerve, muscle, cartilage, and bone tissue are in limited supply for quantitative analysis of the ECM structures, which require relatively large amounts of tissue. However, combining tissues of multiple digits could provide a solution to this. Furthermore, learnings from tissue type-specific protocols can be applied to improve the digit protocol. For instance, cartilage decellularization protocols are currently being developed and optimized in parallel models [36,37]; once refined, these approaches could readily be applied to enhance cartilage evaluation within the digit model, providing a more complete and clinically relevant scaffold.

Future studies should focus on further optimization of decellularization protocols, and the development of recellularization protocols [31]. Standardization of decellularization techniques and assessment methods, such as graft immunogenicity and function, mechanical evaluation [38], and long-term viability, will be essential to achieving recellularized grafts for clinical use. Despite mechanical strength testing results in this study being comparable between groups, and in line with previous findings in the literature, studies of mechanical properties specific to digital tendons are scarce in the literature. Nonetheless, this will be an important outcome to determine the clinical functionality of these grafts. Humanized animal models could be used to assess in vivo function. Furthermore, future investigations could involve transplantation studies comparing decellularized scaffolds with conventional allogeneic transplants. Identifying critical components, such as the necessary level of decellularization and structural preservation, the method of recellularization of complex tissues, thereby accommodating the different tissue types, and how to keep the graft alive during the recellularization process, will be of importance. Additionally, collaborative efforts among researchers, clinicians, and regulatory agencies are necessary to navigate the regulatory landscape and facilitate the translation of recellularized digits into clinical practice, offering off-the-shelf VCAs, and ultimately benefiting patients in need of reconstructive surgery.

## Data Availability

The original contributions presented in this study are included in the article/Supplementary Material. Further inquiries can be directed to the corresponding author(s).

## Supplementary Materials

The following supporting information can be downloaded at: https://www.mdpi.com/article/10.3390/bioengineering12040383/s1. Figure S1: Histological analysis of ECM tissues of human digits. In correspondence to skin analysis, ECM structures in vessels, muscle, and bone tissues show maintenance of intensity in decellularized samples compared to native samples. While some changes in ECM components were found, structural integrity appears relatively preserved. Main alterations found are loss of organization of ECM components such as elastin fibers, collagen fibers, and GAGs. Scale bar represents 100 μm. Figure S2: Stress strain curve analysis of native and decellularized digit tendons. Graphical output of python script specified to output of Instron 5500 Universal Testing Machine. (A) Native digit tendon tensile strength test data. (B) Decellularized digit tendon tensile strength data. Figure S3: Python script for stress strain curve analysis and Young’s Modulus calculation. Figure S4: Tendon-specific mechanical strength testing of decellularized digit tendons. Quantification of tensile stress maximum values and calculated Young’s Modulus values of digital tendons from native and decellularized digits, separated by extensor, superficial flexor, and deep flexor tendons. Video S1: Dynamic functional assessment. Isolated range of motion of (1) superficial flexor, (2 and 4) extensor, and (3) deep flexor tendon are shown. It is demonstrated that tendons have near full range of motion and show isolated function, despite increase in interstitial fluid.
