# Supplementary material for: Decellularization of Human Digits: A Step Towards Off-the-Shelf Composite Allograft Transplantation
Source: Bioengineering (Basel). 2025 Apr 3;12(4):383. doi: 10.3390/bioengineering12040383 (PMC12025325; doi:10.3390/bioengineering12040383)
Supplement: Supplementary file 1 [file bioengineering-12-00383-s001.zip › Supplementary Material_highlights.pdf]

SUPPLEMENTARY MATERIAL

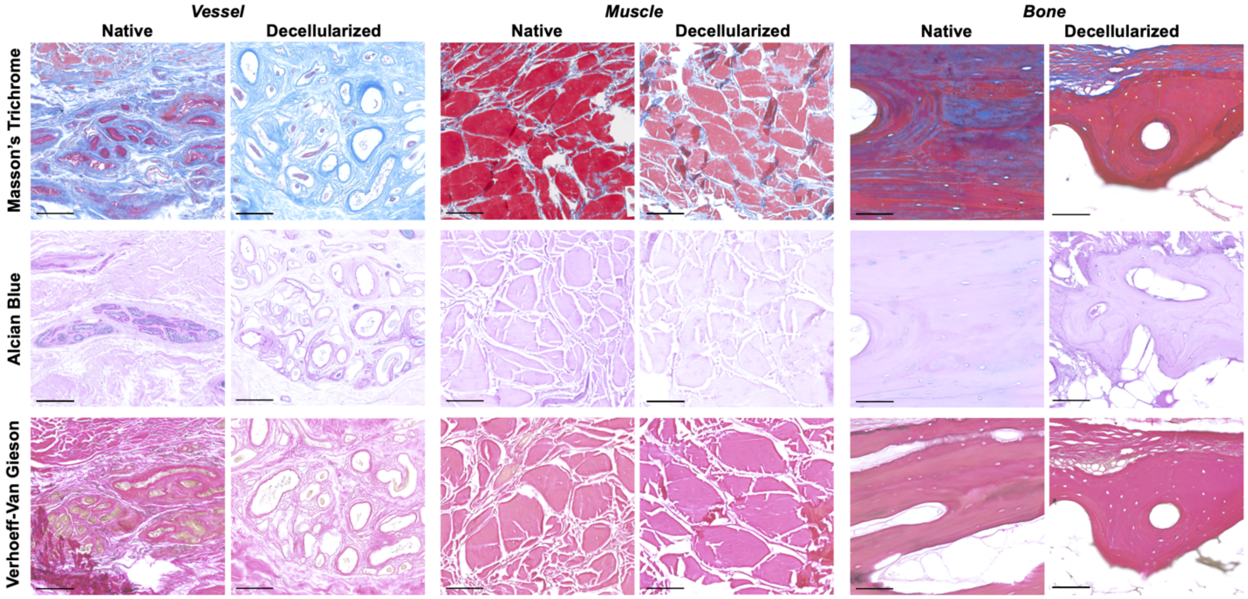

Figure S1. Histological analysis of ECM tissues of the human digits. In correspondence to skin analysis, ECM structures in the vessels, muscle and bone tissues show maintenance of intensity in decellularized samples compared to native. While some changes in ECM components were found, structural integrity appears relatively preserved. Main alterations found are loss of organization of ECM components such as elastin fibers, collagen fibers and GAGs. Scale bar represents 100 µm.

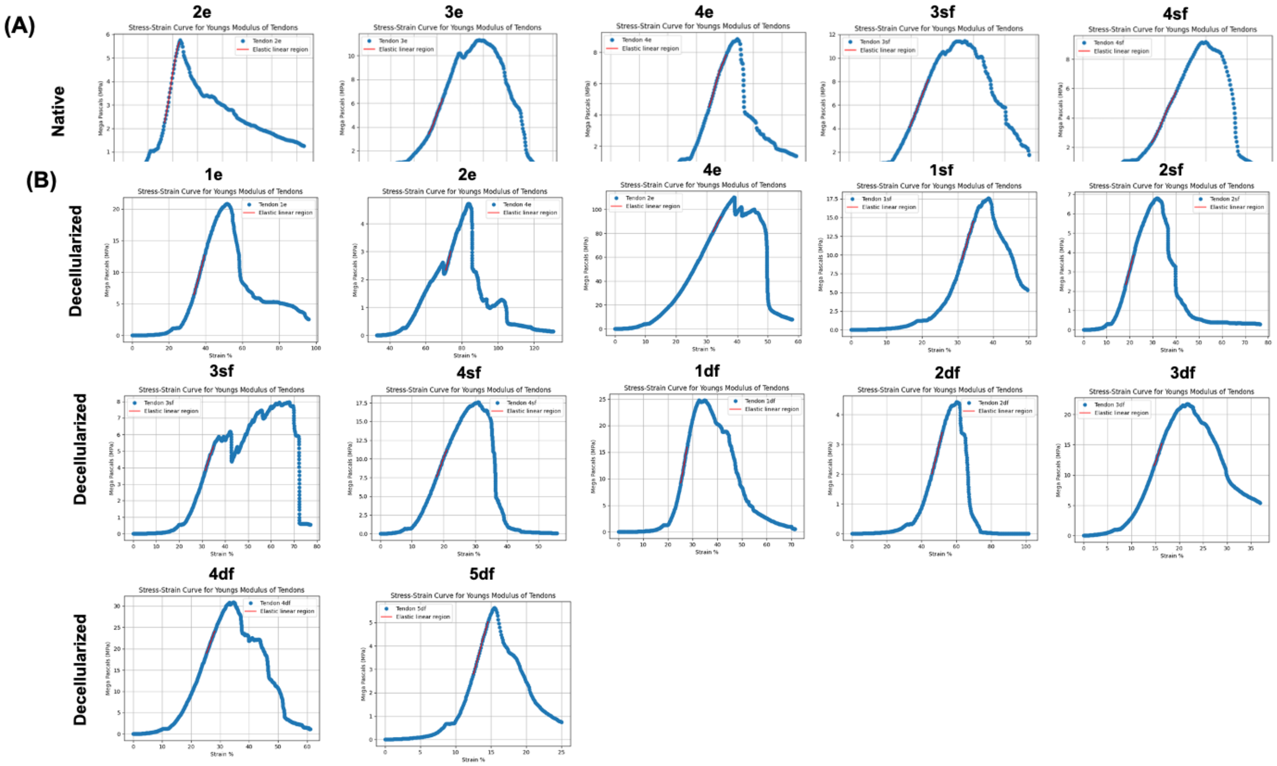

Figure S2. Stress strain curve analysis of native and decellularized digit tendons. Graphical output of python script specified to the output of the Instron 5500 Universal Testing Machine. **(A)** Native digit tendon tensile strength test data **(B)** Decellularized digit tendon tensile strength data.

```
from matplotlib import pyplot as plt
from matplotlib import figure
import numpy as np
from numpy import pi, sin, linspace, exp, polyfit

def calculate_gradient(x1, y1, x2, y2):
    # Calculate the gradient between two points (x1, y1) and (x2, y2)
    if x1 == x2 and y1 < y2:
        return float('inf')  # Handle vertical lines
    return (y2 - y1) / ((x2*0.01) - (x1*0.01)) #As we work with a fraction of 100% strain, x values are multiplies by
0.01.

def find_region_with_greatest_gradient(x_values, y_values):
    if len(x_values) != len(y_values) or len(x_values) < 2:
        raise ValueError("x_values and y_values must be lists of equal length with at least 2 elements.")

    max_gradient = 0
    max_gradient_start = None
    max_gradient_end = None

    for i in range(len(x_values) - 1):
        gradient = calculate_gradient(x_values[i], y_values[i], x_values[i + 1], y_values[i + 1])
        if gradient > max_gradient and gradient > 0:
            max_gradient = gradient
            max_gradient_start = i
            max_gradient_end = i + 1

    return max_gradient, max_gradient_start, max_gradient_end

from openpyxl import load_workbook
# The source xlsx file is named as source.xlsx
wb=load_workbook("_____.xlsx")  #INPUT Directory for Xcell data sets

ws = wb.active
sheet_names = wb.sheetnames

print(sheet_names)
n=0
# Loop through each sheet and process the data
for sheet_name in sheet_names:
    ws = wb[sheet_name]
    stress_data = ws['D'] #D=Force #Collumn letter of stress data in excel
    strain_data = ws['K']    #Collumn letter of strain data in excel
    n = n +1

    print(sheet_name)
# Create the list
    strain_tendon = []
    stress_tendon = []

    strain_tendon = [cell.value for cell in strain_data[1:]]
    stress_tendon= [cell.value for cell in stress_data[1:]]

    strain = []
    stress = []

    for i in range(len(strain_tendon)):
        if strain_tendon[i] == ' ':
            strain_tendon[i] = '0'
        strain.append(np.float64(strain_tendon[i]))

    for i in range(len(stress_tendon)):
        if stress_tendon[i] == ' ':
            stress_tendon[i] = '0'
        stress.append(np.float64(stress_tendon[i]))

    max_gradient, start_index, end_index = find_region_with_greatest_gradient(strain, stress)
    print("The Youngs Modulus for the linear region is =")
    print(max_gradient)
    print("Mega Pascals")

    tendon = sheet_name
    plt.plot(strain, stress, "o", label="Tendon " + tendon)
   # plt.plot(strain[start_index], stress[end_index], "*", color="black")
```

```
    plt.plot(strain[start_index - 10:end_index + 10], stress[start_index - 10:end_index + 10], "-", color="red",
label="Elastic linear region")
    plt.legend(loc=0)
    plt.xlabel("Strain %")
    plt.ylabel("Mega Pascals (MPa)")
    plt.title("Stress-Strain Curve for Youngs Modulus of Tendons")
    plt.grid(True)
    plt.show()
```

Figure S3. Python script for stress strain curve analysis and Youngs' modulus calculation

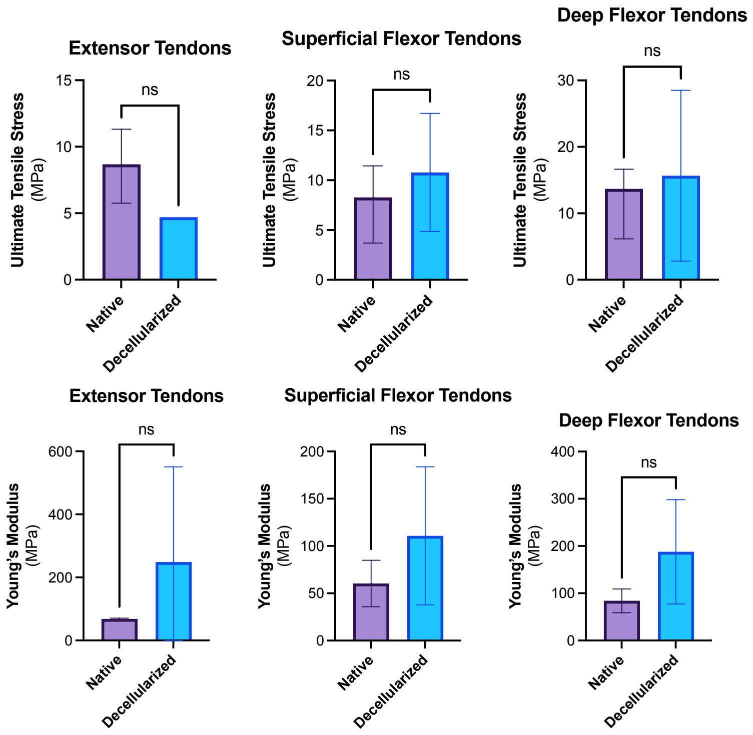

Figure S4. Tendon-specific mechanical strength testing of decellularized digit tendons. Quantification of tensile stress maximum values and the calculated Young's modulus values of digital tendons from native and decellularized digits, separated by the extensor, superficial flexor, and deep flexor tendons.

Video 1. Dynamic functional assessment. Isolated range of motion of the (1) superficial flexor, (2 & 4) extensor, and (3) deep flexor tendon are shown. It is demonstrated, the tendons have a near full range of motion and show isolated function, despite the increase of interstitial fluid.
